# Articulation of Artificial Teeth for Their Better Retention

**Published:** 1884-09

**Authors:** W. E. Driscoll

**Affiliations:** Bedford, Ind.


ARTICLE V.

### ARTICULATION OF ARTIFICIAL TEETH WITH A VIEW TO THEIR BETTER RETENTION IN POSITION.

DR. W. E. DRISCOLL, BEDFORD, IND.

(Read before the Indiana Dental Society.)

Stated in the fewest words, the improvement consists in securing backward pressure upon an upper set of teeth, and forward pressure upon a lower set. No one will, I suppose, question the propriety of this as applied to an upper set. At first view, its application to a lower set seems more open to objection. Press the lower set forward and we utilize the only approach to a rim that can be left to many lower sets, that between the jaws under the molar teeth. Forward pressure makes this rim fit firmly to this part of the gum. A failure to do so is a matter of common complaint with those learning to wear artificial teeth. Such close fitting will produce soreness at first, but in time the parts will toughen the same as any other part of the gums.

How are we to secure the backward pressure upon upper sets and forward pressure on lower sets at the same time? It must be done by elevating the line of articulation at the front teeth as much as can be done with lower teeth extremely short, from the pins to the grinding surfaces of the molars. Teeth must be selected with this object in view, or the plan can not be carried to a successful issue.

Very often, four bicuspids must be left off to shorten the sets so they will allow the necessary depressions upon the posterior portion of the gums. When setting up the teeth, leave a space between the front teeth that will allow an ordinary thickness of blotting paper to pass between their cutting edges when the molars are firmly set on their grinding surfaces. After a few days or weeks wear, the front teeth will be found striking together freely as hard as is desirable, if not so much so as to require some grinding to again throw the main pressure upon the back teeth. The outside and inside cusps of the molars, above and below, must be on an exact level—outside ones no higher than the lingual or palatal ones. When an extreme degree of absorption has taken place, the superior molars will extend near an inch from the upper gums. But let no one hesitate to try them in that way, and the result will be a surprise. Where the inferior alveola is practically absent, care must be taken to set the six front teeth fully as far backward as the ridge should be, and in extremely discouraging cases it will at times be best to set these six teeth a little inside of where the ridge has been. This will, in some cases, leave the upper front teeth standing much more prominent or outside of the line of the lower ones. If this can not be remedied by setting the upper teeth under the gums without interfering with the upward slant of the line of articulation, then the hiatus must be endured, and in many mouths it will be found to amount to no real objection upon trial.

In cases where the short appearance of the upper front teeth would be an objection, an off-set may be made between the molars and bicuspids of each set that will secure or produce the desired backward pressure upon the upper set without the upward slant extending so far forward. This off-set may also be used where there is not room between the ridges to get the amount of dip necessary to a practical result; or to get the upper front teeth to show as desired.

The upper slant of the line of articulation might terminate at the first bicuspids; but this will not be found necessary, as a rule, and in some cases would defeat the attempt to induce the patient to wear teeth at all.

Where a portion of the lower natural teeth are retained, all points of articulation that have a tendency to press the upper plate forward, must be avoided, and all available points to construct slanting surfaces that will result in backward pressure upon the plate must be used. Sometimes this can be done by setting a tooth on each side, so as to receive a glancing stroke from the lower teeth, or prominences of the material of which the plate is made may be so placed as to shut down behind a posterior lower tooth on each side. Sometimes several of the molars are very much elevated above a plane with the teeth anterior to them, and have a very decided tendency to press an upper set out of the mouth. Such teeth, when leaning forward, must not be allowed to strike the upper teeth with their grinding surfaces; but to utilize them in keeping the plate in position, prominences of plate material must extend downward behind them, presenting an inclined plane for them to strike against, that will press the plate backward. Patients may object to these prominences, slanting strokes, etc., at first, but when they realize their value there will be an end to objections.

Some critics may say this plan will require teeth to be made without regard to their natural appearance, or the restoration of sunken features, etc. Not so, if rightly understood and practiced. Although the points of contact of the opposite sets of teeth may be too far inward to restore the expression, this may be counterbalanced by additional material in the rims. And with a full rim and teeth, set well inward, the lips may and do take hold upon these rims and aid to retain them in their place. Very often teeth, instead of being assisted in this way to retain their place upon the gums, are forced from their position by the action of the lips and cheeks.

An accurate bite is necessary when these slanting surfaces and prominences are used in articulating teeth. To secure this, I have an improvement upon the plan mentioned two years ago. When the wax is placed between the gums, direct the patient to swallow at the same moment that the jaws close toward each other. This plan is so satisfactory as to leave nothiug to be desired further in that particular. If a hard substance is embedded in the wax to arrest the closure of the jaws at the right distance from each other, the gum will generally be flattened at that point. So when the models are placed in the bite, the flattened points in the bite must be cut out that the models may go accurately to their proper positions.

In constructing teeth upon the cheap bases, I do not hesitate to say that all trying teeth in the mouth after grinding up and before being finished for use, is worse than useless. There can be no dependence in the way a patient will close the mouth with a loose lot of gutta-percha therein. The length of the teeth, the fullness of the rims, and everything desirable to know can be ascertained by means so much more simple and practical that I do not consider it worth while to go into a description of them.—*Items of Interest.*

